# Predictive value of inflammation-based prognostic scores in patients with metastatic renal cell carcinoma treated with cytoreductive nephrectomy

**DOI:** 10.18632/oncotarget.24507

**Published:** 2018-02-16

**Authors:** Hironori Fukuda, Toshio Takagi, Tsunenori Kondo, Satoru Shimizu, Kazunari Tanabe

**Affiliations:** ^1^ Department of Urology, Tokyo Women's Medical University, Shinjuku-ku, Tokyo, Japan; ^2^ Department of Urology, Tokyo Women's Medical University Medical Center East, Arakawa-ku, Tokyo, Japan; ^3^ Department of Medical Education, Tokyo Women's Medical University, Shinjuku-ku, Tokyo, Japan

**Keywords:** renal cell carcinoma, metastasis, cytoreductive nephrectomy, prognosis, inflammation-based prognostic score

## Abstract

Inflammation-based prognostic scores are useful for predicting survival in various cancers. Here, we aimed to determine the most useful inflammation-based prognostic score for predicting survival in patients with metastatic renal cell carcinoma undergoing cytoreductive nephrectomy. We retrospectively analyzed the data of 152 patients who underwent cytoreductive nephrectomy for metastatic renal cell carcinoma between 1986 and 2015. In the multivariate stepwise analysis, the combination of age, Memorial Sloan-Kettering Cancer Center score, histology, sarcomatoid change, clinical nodal stage, brain metastasis, and liver metastasis was a significant predictor for survival (Harrell's concordance index [c-index]: 0.638). The c-index of the combination improved with the addition of an inflammation-based prognostic score: C-reactive protein (c-index: 0.672), Glasgow prognostic score (c-index: 0.674), neutrophil-to-lymphocyte ratio (c-index: 0.685), lymphocyte-to-monocyte ratio (c-index: 0.670), platelet-to-lymphocyte ratio (c-index: 0.666), systemic inflammation response index (c-index: 0.652), and systemic immune-inflammation index (c-index: 0.678). The neutrophil-to-lymphocyte ratio provided the greatest improvement in the c-index. Additional multivariate analysis showed that the neutrophil-to-lymphocyte ratio was an independent prognostic factor for survival (*P <* 0.0001). The neutrophil-to-lymphocyte ratio was the most useful inflammation-based prognostic score for predicting survival in patients with metastatic renal cell carcinoma treated with cytoreductive nephrectomy.

## INTRODUCTION

Cancers of the kidney and renal pelvis account for approximately 3% of all adult tumors. The American Cancer Society estimated that, in 2017, about 63,990 new cases of kidney cancer (40,610 in men and 23,380 in women) will occur, and that approximately 14,400 people (9,470 men and 4,930 women) will die from this disease [1]. Renal cell carcinoma (RCC) accounts for the majority of kidney and renal pelvis cancers, and approximately 30% of patients with RCC ultimately require systemic therapy for metastatic disease [2].

For patients with metastatic renal cell carcinoma (mRCC), in the cytokine therapy era cytoreductive nephrectomy (CN) of the primary tumor has been shown to improve survival as reported in two randomized trials and a combined analysis [3–5]. After targeted molecular therapies, several agents targeting the vascular endothelial growth factor (VEGF) [6–9] and mammalian target of rapamycin pathways [10, 11], were introduced to mRCC treatment, some retrospective studies suggesting similar survival benefit of CN have existed [12–15], although conflict opinions also have been reported [16]. These controversial results are currently being evaluated by two ongoing randomized trials (CARMENE and SURTIME). In either case, because not all patients with mRCC will benefit from CN, prognostic factors affecting mRCC patients should be investigated to optimize the benefits of CN.

It was previously reported that various inflammation-based prognostic scores, such as C-reactive protein (CRP), Glasgow prognostic score (GPS), platelet-to-lymphocyte ratio (PLR), and neutrophil-to-lymphocyte ratio (NLR) might be useful for predicting survival in patients with a malignant neoplasm [17–20]. In addition, CRP, GPS, NLR, the systemic inflammation response index (SIRI), and the systemic immune-inflammation index (SII) have been investigated in patients with mRCC, and have been shown to have a prognostic significance [21–25]. However, it is not clear which of these inflammation-based prognostic scores best predicts survival in patients with mRCC treated with CN.

The aim of the present study was to investigate and compare the predictive accuracy of these various inflammation-based prognostic scores to identify the most useful predictive factor in patients with mRCC treated with CN.

## RESULTS

### Patient characteristics and inflammation-based prognostic scores

Table 1 shows the patient characteristics of the 152 patients with mRCC who were treated with CN. Because all of these patients had synchronous metastasis at the time of RCC diagnosis, there were no patients with a favorable Memorial Sloan-Kettering Cancer Center (MSKCC) risk score; 106 patients (70%) were classified as intermediate risk and 46 patients (30%) were classified as poor risk. The treatment details for metastasis existing at CN were described in Table 2. Furthermore, the values of the various inflammatory prognostic factors are shown in Table 3, including CRP, GPS, NLR, LMR, PLR, SIRI, and SII.

**Table 1:** Patient characteristics (*N* = 152)

Age, years	Median (95% CI)	64.0 (61.5–64.8)
Sex		
Male	*n* (%)	109 (72)
Female	*n* (%)	43 (28)
ECOG-PS		
0	*n* (%)	89 (59)
1	*n* (%)	43 (28)
2	*n* (%)	14 (9)
3	*n* (%)	6 (4)
MSKCC risk		
Intermediate	*n* (%)	106 (70)
Poor	*n* (%)	46 (30)
Histology		
Clear cell carcinoma	*n* (%)	138 (91)
Non-clear cell carcinoma	*n* (%)	14 (9)
Sarcomatoid change	*n* (%)	22 (14)

Abbreviations: ECOG-PS, Eastern Cooperative Oncology Group performance status; MSKCC, Memorial Sloan-Kettering Cancer Center.

**Table 2:** Treatment for metastasis existing at cytoreductive nephrectomy

Systemic treatment, *n* (%)	
Sunitinib	36 (24)
Sorafenib	16 (11)
Temsirolimus	4 (3)
Pazopanib	5 (3)
Axitinib	2 (1)
Interferon	52 (34)
IL2	5 (3)
Interfero*n* + IL2	2 (1)
Metastasectomy, *n* (%)	6 (4)
EBRT, *n* (%)	6 (4)
None, *n* (%)	14 (9)
Unknown, *n* (%)	4 (3)

Abbreviations: IL2, interleukin-2; EBRT, external beam radiation therapy.

**Table 3:** The value of the inflammation-based prognostic scores

CRP, mg/dl	Median (95% CI)	2.4 (3.9–5.7)
GPS		
0	*n* (%)	47 (31)
1	*n* (%)	59 (39)
2	*n* (%)	46 (30)
NLR	Median (95% CI)	3.2 (1.1–10.8)
LMR	Median (95% CI)	3.4 (3.5–4.2)
PLR	Median (95% CI)	195 (200–233)
SIRI		
0	*n* (%)	29 (19)
1	*n* (%)	68 (45)
2	*n* (%)	55 (36)
SII	Median (95% CI)	819 (920–1168)

Abbreviations: CRP, C-reactive protein; GPS, Glasgow prognostic score; NLR, neutrophil to lymphocyte ratio; LMR, lymphocyte to monocyte ratio; PLR, platelet to lymphocyte ratio; SIRI, systemic inflammation response index; SII, systemic immune-inflammation index.

### The relationship between clinicopathologic factors including inflammation-based prognostic scores and overall survival in patients with mRCC treated with CN

During the follow-up period, 92 patients (61%) died of various causes and 84 patients (55%) died of RCC. The results of the univariate analysis for overall survival (OS) are shown in Tables 4 and 5. Table 4 shows that Eastern Cooperative Oncology Group performance status (ECOG-PS) (*P* = 0.0006), MSKCC risk (*P <* 0.0001), histology (*P* = 0.0099), sarcomatoid change (*P* = 0.0022), number of metastatic organs (*P* = 0.011), brain metastasis (*P* = 0.037), and liver metastasis (0.0015) were significantly associated with OS. Table 5 shows that all inflammation-based prognostic scores were significantly associated with OS.

**Table 4:** Univariate and multivariate stepwise analysis of prognostic factors other than inflammation-based prognostic scores for overall survival in patients with metastatic renal cell carcinoma treated with cytoreductive nephrectomy

	Univariate analysis HR	95% CI	*P* value	Stepwise analysis HR	95% CI	*P* value
Age	1.02	0.999–1.04	0.064	1.03	1.009–1.057	0.0062
Sex			0.27	-	-	-
Male	1.00	Reference		-	-	-
Female	1.29	0.816–1.98		-	-	-
ECOG-PS			0.0006	-	-	-
0,1	1.00	Reference		-	-	-
≥2	2.83	1.61–4.70		-	-	-
MSKCC risk			<0.0001			<0.0001
Intermediate	1.00	Reference		1.00	Reference	
Poor	2.50	1.63–3.79		2.65	1.71–4.08	
Histology			0.0099			0.0008
Clear cell carcinoma	1.00	Reference		1.00	Reference	
Non-clear cell carcinoma	2.74	1.30–5.23		4.28	1.91–8.83	
Sarcomatoid change	2.59	1.44–4.39	0.0022	3.83	2.06–6.81	
Clinical T stage			0.23			-
cT1-2	1.00	Reference		-	-	-
cT3-4	1.40	0.817–2.58		-	-	-
Clinical nodal stage			0.17			0.85
N0	1.00	Reference		1.00	Reference	
N1	0.779	0.272–1.76		0.84	0.290–1.91	
N2	1.57	0.934–2.53			0.643–1.83	
Primary tumor size	1.006	1.00–1.01	0.050	-	-	-
Number of metastatic organs			0.011			-
1	1.00	Reference		-	-	-
≥2	1.78	1.15–2.72		-	-	-
Brain metastasis	3.65	1.10–8.99	0.037	6.97	2.00–18.6	0.0046
Liver metastasis	3.89	1.77–7.64	0.0015	4.42	1.95–9.10	0.0008

Abbreviations: HR, hazard ratio; CI, confidence interval; ECOG-PS, Eastern Cooperative Oncology Group performance status; MSKCC, Memorial Sloan-Kettering Cancer Center.

**Table 5:** Univariate analysis of inflammation-based prognostic scores for overall survival in patients with metastatic renal cell carcinoma treated with cytoreductive nephrectomy

Univariate analysis	HR	95% CI	*P* value
CRP	1.08	1.05–1.12	<0.0001
GPS			<0.0001
0	1.00	Reference	
1	1.89	1.12–3.21	
2	3.54	2.07–6.14	
NLR	1.32	1.19–1.45	<0.0001
LMR	0.814	0.717–0.914	0.0003
PLR	1.0004	1.0002–1.0004	<0.0001
SIRI			0.0004
0	1.00	Reference	
1	3.30	1.70–7.21	
2	3.42	1.69–7.67	
SII	1.0006	1.0003–1.0009	<0.0001

Abbreviations: HR, hazard ratio; CI, confidence interval; CRP, C-reactive protein; GPS, Glasgow prognostic score; NLR, neutrophil to lymphocyte ratio; LMR, lymphocyte to monocyte ratio; PLR, platelet to lymphocyte ratio; SIRI, systemic inflammation response index; SII, systemic immune-inflammation index.

### NLR improved the predictive accuracy for overall survival in patients with mRCC treated with CN to a greater extent than did other inflammation-based prognostic scores

**Figure 1:**
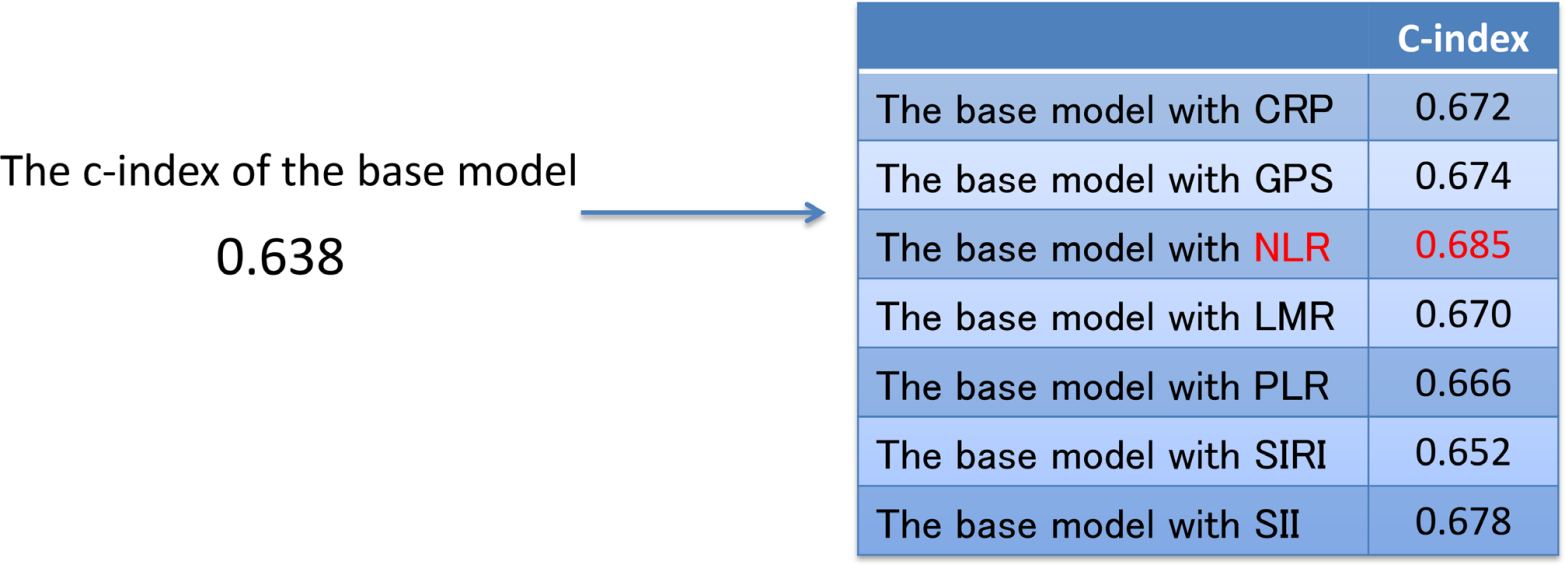
The c-index of the base model and the base model with each inflammation-based prognostic score Abbreviations: c-index, concordance index; CRP, C-reactive protein; GPS, Glasgow prognostic score; NLR, neutrophil-to-lymphocyte ratio; LMR, lymphocyte-to-monocyte ratio; PLR, platelet-to-lymphocyte ratio; SIRI, systemic inflammation response index; SII, systemic immune-inflammation index.

Multivariate stepwise Cox's proportional hazards model analysis revealed that the best combination of prognostic factors for OS, excluding inflammation-based prognostic scores, included the following 7 factors (base model): age, MSKCC, histology, sarcomatoid change, clinical nodal stage, brain metastasis, and liver metastasis (Table 4). To evaluate the predictive accuracy for OS, the c-index was calculated. The c-index in the base model was 0.638. The c-index was improved by the addition of CRP (c-index: 0.672), GPS (c-index: 0.674), NLR (c-index: 0.685), LMR (c-index: 0.670), PLR (c-index: 0.666), SIRI (c-index: 0.652), and SII (c-index: 0.678). NLR improved the c-index to a greater extent than did the other inflammation-based prognostic scores (Figure 1). Multivariate analysis using the base model and NLR revealed that NLR was an independent prognostic factor for OS (*P <* 0.0001) (Table 6).

**Table 6:** Multivariate analysis of base model with neutrophil to lymphocyte ratio for overall survival in patients with metastatic renal cell carcinoma treated with cytoreductive nephrectomy

Multivariate analysis	HR	95% CI	*P* value
Age	1.03	1.006–1.054	0.014
MSKCC risk			0.0003
Intermediate	1.00	Reference	
Poor	2.38	1.51–3.72	
Histology			0.0016
Clear cell carcinoma	1.00	Reference	
Non-clear cell carcinoma	3.86	1.73–7.91	
Sarcomatoid change	3.24	1.74–5.77	0.0004
Clinical nodal stage			0.18
N0	1.00	Reference	
N1	0.44	0.141–1.09	
N2	1.11	0.646–1.82	
Brain metastasis	7.46	2.15–19.9	0.0035
Liver metastasis	6.25	2.70–13.3	<0.0001
NLR	1.33	1.19–1.49	<0.0001

Abbreviations: HR, hazard ratio; CI, confidence interval; MSKCC, Memorial Sloan-Kettering Cancer Center; NLR, neutrophil to lymphocyte ratio.

### Analysis of the ideal cutoff value of the NLR

**Figure 2:**
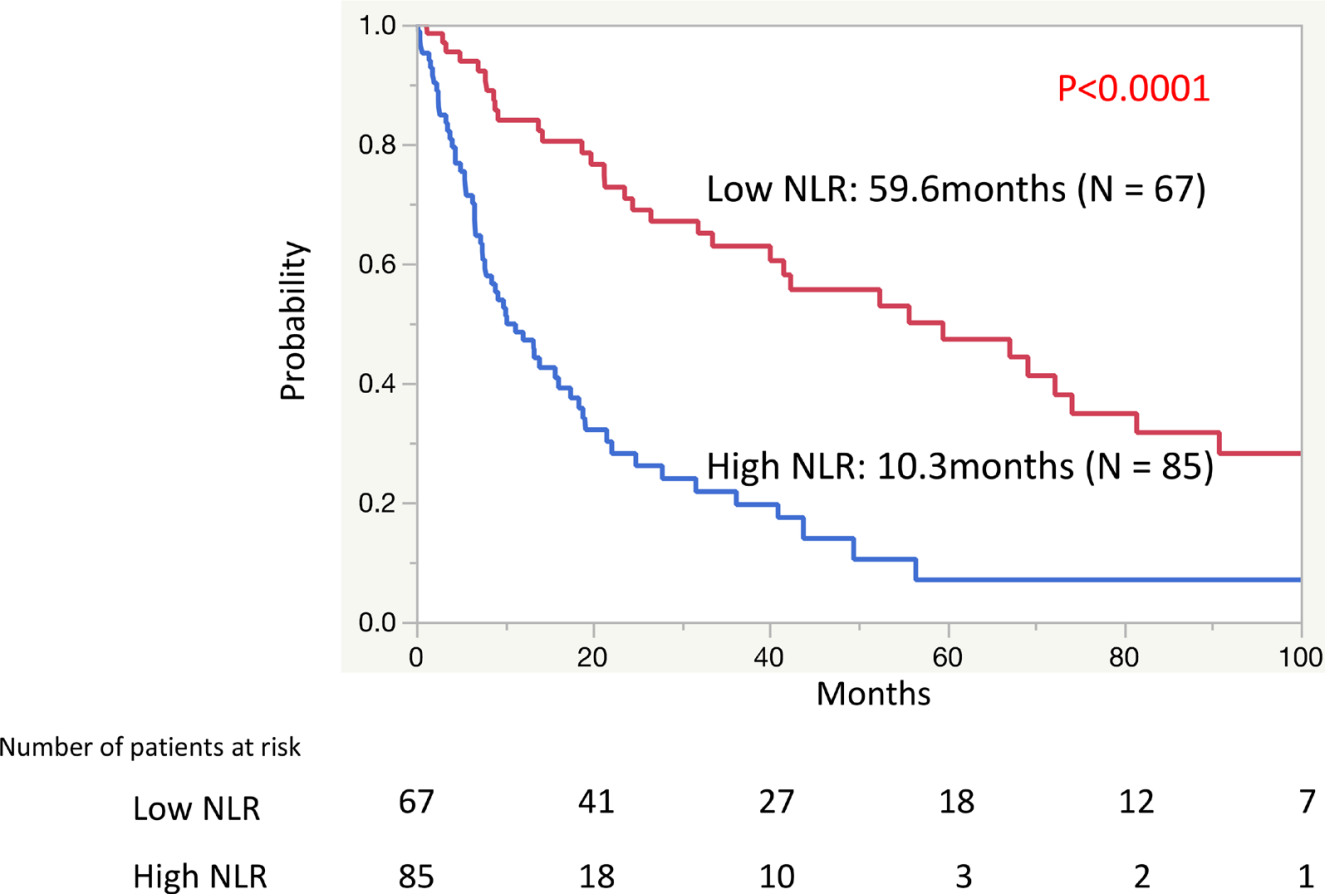
The Kaplan-Meier curves of the overall survival rates between patients with a low neutrophil-to-lymphocyte ratio (NLR) and a high NLR

To detect the ideal cutoff value of NLR for OS in patients with mRCC treated with CN, receiver operating characteristic (ROC) analysis was performed using the mortality at 40 months after CN, resulting in an NLR value of 3.15. To simplify, we approximated the ideal cutoff value of NLR to a value of 3.0. There were 67 patients with a low NLR value (< 3.0) and 85 patients with a high NLR value (≥ 3.0). The Kaplan-Meier curves showed a significant difference in the OS rates between patients with a low NLR (median: 59.6 months) and a high NLR (median: 10.3 months) (*P <* 0.0001) (Figure 2).

## DISCUSSION

The present study investigated various predictors, including inflammation-based prognostic scores, of survival in patients with mRCC who underwent CN. Our findings demonstrate that NLR is the most effective factor among inflammation-based prognostic scores for improving the predictive accuracy of factors related to patient and tumor characteristics.

Although primary tumor resection with CN has been shown to improve survival in several previous studies of patients with mRCC [12, 13], the surgical procedure is invasive and has potential risks. To determine the best candidates for CN, prognostic factors for survival in patients with mRCC treated with CN should be considered. Numerous studies have previously reported that inflammation-based prognostic scores might be useful for predicting survival in patients with various malignancies. The GPS is a selective combination of CRP and albumin serum levels that has been examined and validated in more than 60 studies for a variety of cancers [17, 26]. The PLR has been identified as an independent prognostic marker for survival in breast cancer patients. Elevated preoperative PLR levels have been associated with cause-specific survival in univariate analysis (hazard ratio [HR]: 2.75, 95% confidence interval [CI]: 1.57–4.83, *P <* 0.001) and multivariate analysis (HR: 2.03, 95% CI: 1.03–4.02, *P* = 0.042) [18].

Additionally, the usefulness of inflammation-based prognostic scores has been reported in patients with mRCC. In patients with mRCC treated with first line sunitinib therapy, patients with a low SII had a significantly longer OS than did those with a high SII (median OS: 43.6 months vs. 13.5 months, *P <* 0.0001) [25]. In patients with mRCC treated with CN, the SIRI appeared to be an independent prognostic factor for OS and was significantly associated with aggressive tumor behavior [23]. Despite these studies, it is not clear which inflammation-based prognostic scores best predict survival in patients with mRCC treated with CN.

The present study identified NLR as the best predictor among the various inflammation-based prognostic scores that we evaluated for predicting survival in patients with mRCC who underwent CN. Patients with a low NLR (<3.0) had a significantly longer OS than did those patients with a high NLR (≥3.0) (median OS: 59.6 months vs. 10.3 months, respectively) (*P <* 0.0001). The usefulness of NLR for predicting survival has been reported in many previous studies of various cancers [20, 27–30]. In patients with RCC, the preoperative NLR levels in patients with a localized nonclear-cell RCC was shown to be significantly associated with disease-free survival in univariate analysis (HR: 1.15, *P* = 0.028) and in multivariate analysis (HR: 1.17, *P* = 0.022) [31]. In patients with mRCC treated with CN, it was reported that a high NLR (≥4.0) was significantly associated with poor outcomes. The median OS of the patients with high NLR in that study (≥4.0) was 10.2 months, which was significantly shorter than that of the patients with a low NLR (<4.0) (36.5 months, *P* = 0.002) [24].

The association between inflammation and the cancer progression has been investigated in many previous studies. NLR is a marker of inflammation and immunity. Neutrophils promote angiogenesis and inhibit the anti-tumor immune system response, resulting in tumor growth [32, 33]. VEGF, secreted by neutrophils, induces angiogenesis and promotes tumor growth, recurrence, invasion, and metastasis [34, 35]. On the other hand, lymphocytes are essential in tumor defense. Lymphocytes can elicit cytotoxic cell death and interfere with tumor cell proliferation and migration [27]. In patients with RCC, high levels of lymphocytic attractant chemokine expression have been shown to be a favorable prognostic factor [36]. Therefore, a high NLR, which reflects an increased neutrophil count or a decreased lymphocyte count, can be a useful prognostic factor in cancer patients.

There are some limitations of the present study, including its retrospective and single-center study design. In addition, we were not able to assess the data of all inflammation-based prognostic scores in all patients because some scores were unknown or not measured. Nevertheless, the present study, to our best knowledge, is the first study investigating which inflammation-based prognostic score can best predict survival in patients with mRCC treated with CN. Future large-scale prospective multi-center studies are needed to confirm our findings.

## MATERIALS AND METHODS

### Patients

After approval by the institutional review board, the present study retrospectively reviewed the medical records of patients at our hospital and identified 152 patients diagnosed with mRCC who were treated with CN between March 1986 and August 2015. The median follow-up period was 14 months, and the survival data was collected until 100 months after CN. Tumor stage was determined according to the 2009 TNM classification [37]. Pathological diagnoses were made according to the 2016 World Health Organization classification [38]. Stratification of prognostic risk was done according to the MSKCC risk classification [39].

### Measurements and definitions

Clinical, laboratory, and survival data were collected by reviewing the electronic medical records of the patients. Pathologic data were obtained from nephrectomy specimens. Surgical specimens were processed according to standard pathological procedures. All specimens were histologically confirmed to be RCC.

We examined the seven inflammation-based prognostic scores, including CRP, GPS, NLR, LMR, PLR, SIRI, and SII. The GPS was calculated as previously described [17]. Briefly, patients with an elevated CRP concentration (>1.0 mg/dL) and a decreased albumin concentration (<3.5 g/dL) were assigned a score of 2. Patients with an elevated CRP concentration (>1.0 mg/dL) or a decreased albumin concentration (<3.5 g/dL) were assigned a score of 1, and patients with a CRP concentration of ≤1.0 mg/dL and an albumin concentration of ≥3.5 g/dL were assigned a score of 0 (Table 7A). The NLR was defined as the serum absolute neutrophil count divided by the lymphocyte count in the peripheral blood [40]. The LMR was defined as the serum absolute lymphocyte count divided by the monocyte count in the peripheral blood [41]. The PLR was calculated as the absolute platelet count measured in ×10^9^ L^−1^ divided by the absolute lymphocyte count measured in × 10^9^ L^−1^ [18]. The SIRI was defined as follows: patients with both elevated hemoglobin and elevated LMR (≥ 137/116 gL^−1^ and ≥ 3.23, respectively) were allotted to group 0; patients with either elevated hemoglobin or elevated LMR were allotted to group 1; and patients with both a decreased hemoglobin and a decreased LMR (< 137/116 gL^−1^ and < 3.23, respectively) were assigned to group 2 (Table 7B) [23]. The SII was defined as follows: SII = P × N/L, where P, N, and L were the preoperative peripheral platelet, neutrophil, and lymphocyte counts, respectively [25, 42].

**Table 7:** (A) Glasgow prognostic score

	Points
C-reactive protein ≤ 1.0 mg/dl and albumin ≥ 3.5 g/dl	0
C-reactive protein >1.0 mg/dl or albumin < 3.5 g/dl	1
C-reactive protein >1.0 mg/dl and albumin <3.5 g/dl	2

**Table:** (B) Systemic inflammation response index

	Points
Hemoglobin ≥ 137/116 gl^−1^ (male/female) and LMR ≥ 3.23	0
Hemoglobin ≥ 137/116 gl^−1^ (male/female) or LMR ≥ 3.23	1
Hemoglobin < 137/116 gl^−1^ (male/female) and LMR < 3.23	2

Abbreviation: LMR, lymphocyte to monocyte ratio.

### Statistical analysis

Survival analysis was performed using the Cox's proportional hazards model. Multivariate stepwise Cox's proportional hazards model analysis was performed to select the best combination of prognostic factors. The predictive accuracy was evaluated using Harrel's concordance index (c-index) [43]. A ROC curve was performed to estimate the optimal cut-off value. OS curves were estimated using the Kaplan-Meier method and compared using the log-rank test. A difference was considered significant at *P <* 0.05. Statistical analyses were performed using JMP 11.0.0 (SAS Institute, Cary, NC, USA) and SAS v.9.4 (SAS Institute, Cary, NC, USA).

## Abbreviations

c-index: concordance index
CI: confidence interval
CN: cytoreductive nephrectomy
CRP: C-reactive protein
GPS: Glasgow prognostic score
HR: hazard ratio
mRCC: metastatic renal cell carcinoma
MSKCC: Memorial Sloan-Kettering Cancer Center
NLR: neutrophil-to-lymphocyte ratio
OS: overall survival
PLR: platelet-to-lymphocyte ratio
RCC: renal cell carcinoma
ROC: receiver operating characteristics
SII: systemic immune-inflammation index
SIRI: systemic inflammation response index
VEGF: vascular endothelial growth factor
